# Unlocking Biomarker Discovery: Large Scale Application of Aptamer Proteomic Technology for Early Detection of Lung Cancer

**DOI:** 10.1371/journal.pone.0015003

**Published:** 2010-12-07

**Authors:** Rachel M. Ostroff, William L. Bigbee, Wilbur Franklin, Larry Gold, Mike Mehan, York E. Miller, Harvey I. Pass, William N. Rom, Jill M. Siegfried, Alex Stewart, Jeffrey J. Walker, Joel L. Weissfeld, Stephen Williams, Dom Zichi, Edward N. Brody

**Affiliations:** 1 SomaLogic, Boulder, Colorado, United States of America; 2 Department of Pathology, University of Pittsburgh School of Medicine, University of Pittsburgh Cancer Institute, Pittsburgh, Pennsylvania, United States of America; 3 University of Colorado Cancer Center, University of Colorado at Denver, Anschutz Medical Campus, Aurora, Colorado, United States of America; 4 Department of Molecular, Cellular, and Developmental Biology, University of Colorado, Boulder, Colorado, United States of America; 5 Denver Veterans Affairs Medical Center, Denver, Colorado, United States of America; 6 Langone Medical Center and Cancer Center, New York University School of Medicine, New York, New York, United States of America; 7 Division of Pulmonary, and Critical Care, and Sleep Medicine, New York University School of Medicine, New York, New York, United States of America; 8 Department of Pharmacology and Chemical Biology, University of Pittsburgh School of Medicine, University of Pittsburgh Cancer Institute, Pittsburgh, Pennsylvania, United States of America; 9 Department of Epidemiology, University of Pittsburgh Graduate School of Public Health, University of Pittsburgh Cancer Institute, Pittsburgh, Pennsylvania, United States of America; Florida International University, United States of America

## Abstract

**Background:**

Lung cancer is the leading cause of cancer deaths worldwide. New diagnostics are needed to detect early stage lung cancer because it may be cured with surgery. However, most cases are diagnosed too late for curative surgery. Here we present a comprehensive clinical biomarker study of lung cancer and the first large-scale clinical application of a new aptamer-based proteomic technology to discover blood protein biomarkers in disease.

**Methodology/Principal Findings:**

We conducted a multi-center case-control study in archived serum samples from 1,326 subjects from four independent studies of non-small cell lung cancer (NSCLC) in long-term tobacco-exposed populations. Sera were collected and processed under uniform protocols. Case sera were collected from 291 patients within 8 weeks of the first biopsy-proven lung cancer and prior to tumor removal by surgery. Control sera were collected from 1,035 asymptomatic study participants with ≥10 pack-years of cigarette smoking. We measured 813 proteins in each sample with a new aptamer-based proteomic technology, identified 44 candidate biomarkers, and developed a 12-protein panel (cadherin-1, CD30 ligand, endostatin, HSP90α, LRIG3, MIP-4, pleiotrophin, PRKCI, RGM-C, SCF-sR, sL-selectin, and YES) that discriminates NSCLC from controls with 91% sensitivity and 84% specificity in cross-validated training and 89% sensitivity and 83% specificity in a separate verification set, with similar performance for early and late stage NSCLC.

**Conclusions/Significance:**

This study is a significant advance in clinical proteomics in an area of high unmet clinical need. Our analysis exceeds the breadth and dynamic range of proteome interrogated of previously published clinical studies of broad serum proteome profiling platforms including mass spectrometry, antibody arrays, and autoantibody arrays. The sensitivity and specificity of our 12-biomarker panel improves upon published protein and gene expression panels. Separate verification of classifier performance provides evidence against over-fitting and is encouraging for the next development phase, independent validation. This careful study provides a solid foundation to develop tests sorely needed to identify early stage lung cancer.

## Introduction

Lung cancer is the leading cause of cancer deaths, because ∼84% of cases are diagnosed at an advanced stage [1]–[3]. Worldwide in 2008, ∼1.5 million people were diagnosed and ∼1.3 million died [4] – a survival rate unchanged since 1960. However, patients diagnosed at an early stage and have surgery experience an 86% overall 5-year survival [2], [3]. New diagnostics are therefore needed to identify early stage lung cancer.

Over the past decade the clinical utility of low-dose CT has been evaluated [5]–[8] with the hope that high-resolution imaging can help detect lung cancer earlier and improve patient outcomes, much as screening has done for breast and colorectal cancers [9]. Definitive conclusions about CT screening and lung cancer mortality await results from randomized trials in the US [8] and Europe [10]–[13]. CT can detect small, early-stage lung tumors, but distinguishing rare cancers from common benign conditions is difficult and has led to unnecessary procedures, radiation exposure, anxiety, and cost [6], [14]–[16]. We (J.M.S., J.L.W., and colleagues) recently reported such conclusions for the Pittsburgh Lung Screening Study (PLuSS), the largest single-institution CT screening study reported to date [5].

Other types of biomarkers have also been sought [17]. Proteins are attractive because they are an immediate measure of phenotype, in contrast to DNA which provides genotype, largely a measure of disease risk [18]. Single protein biomarkers are the foundation of molecular diagnostics in the clinic today. It is widely thought that multiple biomarkers could improve the sensitivity and specificity of diagnostic tests, and that complex diseases like cancer change the concentrations of multiple proteins [19]. However, discovering multiple protein biomarkers by measuring many proteins simultaneously (proteomics) in complex samples like blood has proven difficult for reasons of coverage, precision, throughput, preanalytical variability, and cost [20].

To enable biomarker discovery, we developed a new proteomic technology that is based on a new generation of aptamer protein binding reagents and has potentially broad application [18]. The current assay measures 813 diverse human proteins in just 15 µL of blood with low limits of detection (1 pM average and as low as 100 fM), 7 logs of overall dynamic range, and high reproducibility (5% median coefficient of variation) [18]. Here we present the first large scale clinical application of our proteomics technology to discover blood protein biomarkers in a large multi-center case-control study conducted in archived samples from 1,326 subjects from four independent studies of non-small cell lung cancer (NSCLC) in long-term tobacco-exposed populations.

## Materials and Methods

### Ethics Statement

All samples were collected from study participants after obtaining written informed consent under clinical research protocols approved by the following institutional review boards: The University of Pittsburgh Institutional Review Board (Pitt); The New York University School of Medicine Institutional Review Board (NYU); The Roswell Park Cancer Institute Institutional Review Board (RP); and The Cape Cod Healthcare Institutional Review Board (BS).

### Study Design

**Figure 1:**
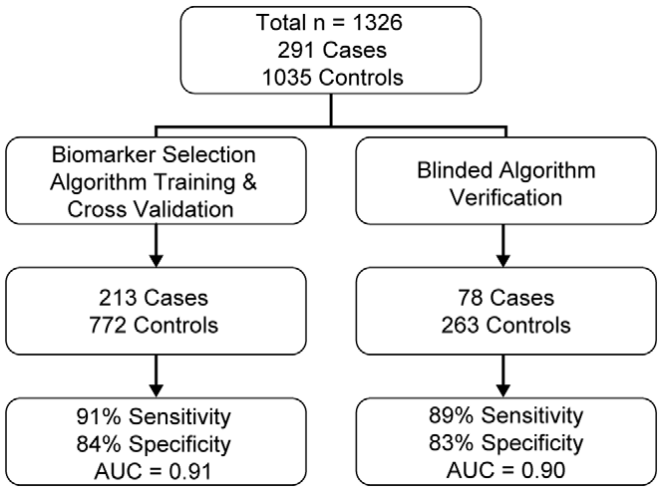
Study Flow for Algorithm Training and Verification.

The objectives of this study were to discover biomarkers that discriminate NSCLC from smokers with ≥10 years of cigarette smoking history, to train and cross-validate a multi-biomarker classifier of NSCLC to meet pre-specified performance criteria, and to verify the performance of this classifier with a separate set of blinded samples. The overall design of the study is shown in Figure 1. We designed and executed this study to current rigorous standards for biomarker clinical studies [21]–[23] with the goals of maximize biomarker robustness, validity, and reliability at the discovery phase, and minimizing potential effects of preanalytical variability. The study was a discovery-phase, case-control design. Critical study design features include the following. The clinical question and study design were pre-specified prior to identifying and acquiring samples. Samples were acquired from four independent study sites in order to control for potential preanalytical variability. Strict standard operating procedures were followed to ensure sample and data anonymity and blinding at all times (see below). A verification sample set consisting of 25% of all samples in the study was randomly selected and the identification of this set was blinded. The statistical analysis plan was pre-specified and included minimally acceptable performance criteria for sensitivity and specificity.

### Sample Cohort

The sample cohort comprised 1,326 serum samples obtained from four independent biorepositories: New York University (NYU) [24]; Roswell Park Cancer Institute (RPCI) [25]; The University of Pittsburgh (PITT) [5]; and a commercial biorepository (BioServe (BS)) (Table 1). All samples were collected from study participants after obtaining informed consent under institutionally approved clinical research protocols as described [5], [24], [25]. Both case and control serum samples were collected from four study centers. The clinical characteristics of the study cohort for the training and verification sets are shown in Table 2. The staging and histology of NSCLC cases is shown in Table 3. The sample cohort included patients diagnosed with pathologic or clinical stage I-III NSCLC and a high-risk control population with a history of long-term tobacco use, including active and ex-smokers with ≥10 pack-years of cigarette smoking. The control populations were selected randomly within each study to represent the patient population at risk for lung cancer that would be candidates for CT screening, with a ratio of case:control of 1∶3.5. Blood samples for cases were collected from patients within eight weeks of the first biopsy-proven lung cancer diagnosis and prior to removal of the tumor by a surgical procedure. All cases used in this study were confirmed as primary lung cancer by pathology review. NSCLC staging was assigned by pathological staging for 240 subjects and clinical staging for 51 subjects. Benign nodule controls have at least one year of follow-up data and non-malignant diagnosis. Smoker controls were asymptomatic study participants with ≥10 pack-years of cigarette smoking. Smoker controls from NYU and Pitt were nodule free by CT; nodule status is unknown for the smoker controls from RP and BS. Demographic data was collected by self-report questionnaires. Additional data for cases was acquired through clinical chart review. Pulmonary function testing was assessed by spirometry for a subset of the study participants.

**Table 1:** Sample cohort by independent study site.

Site	Cases (n = 291)	Nodule Controls (n = 565)	Smoker Controls (n = 470)	Total/Site
BS	43	0	63	106
RPCI	72	66	110	248
NYU	88	238	172	498
PITT	88	261	125	474

**Table 2:** Clinical characteristics of NSCLC case and control sets for training and verification.

		Training Set (n = 985)			Verification Set (n = 341)		
		Cases	Controls	p-value§	Cases	Controls	p-value§
Individuals, no. (%)		213 (21.6)	772 (78.4)		78 (22.9)	263 (77.1)	
Sex (%)	Male	51.2	47.4		43.6	47.9	
	Female	48.8	52.6	0.3305	56.4	52.1	0.5015
Age, mean (SD)		67.6 (9.8)	59.0 (10.2)	<0.0001	68.3 (10.2)	58.8 (9.6)	<0.0001
Control Nodule Status, no. (%)	Benign nodule	n/a	420 (54.4)		n/a	145 (55.1)	
	No nodule	n/a	222 (28.8)		n/a	75 (28.5)	
	Unknown	n/a	130 (16.8)		n/a	43 (16.4)	
Smoking Status, no.	Current	54	421	<0.0001	25	150	<0.0001
	Ex	85	310	<0.0001	31	108	<0.0001
	Never	11	6	<0.0001	7	3	<0.0001
	Unknown	63	35	<0.0001	15	2	<0.0001
Smoking (PKY), mean (SD)‡		47.1 (33.7)	42.3 (24.2)	0.0258	40.9 (30.8)	42.3 (24.6)	0.7003

§ For continuous data the differences were tested using t-tests. For categorical data significant differences were tested using the Pearson Chi-Squared Test for independence.

‡ Pack-years: product of the self reported number of packs of cigarettes smoked per day and the number of years of smoking.

**Table 3:** Clinical characteristics of NSCLC cases in the training and verification sets.

		Training Cases, n = 213, no. (%)	Verification Cases, n = 78, no. (%)
Stage NSCLC§	I	99 (46.5)	38 (49)
	II	32 (15.0)	11 (14)
	III	82 (38.5)	27 (35)
	Not reported	-	2 (2)
Histology	Adenocarcinoma	120 (56.3)	49 (62.8)
	Squamous	71 (33.3)	18 (23.1)
	Large cell	2 (1.0)	2 (2.6)
	NSCLC NOS	20 (9.4)	9 (11.5)

§ Clinical staging for 17 Stage I, 5 Stage II and 29 Stage III cases, NOS not otherwise specified.

### Serum Collection, Processing, Storage, and Shipment

All serum specimens were collected following uniform protocols recommended by the National Cancer Institute's Early Detection Research Network [22]. Three of the centers (NYU, PITT and RPMC) collected serum in red top Vacutainer tubes (Becton Dickinson, Raritan, NJ) and one center (BS) collected serum in tiger top SST Vacutainer tubes (Becton Dickinson). All samples were allowed to clot and serum was recovered by centrifugation within 2–8 hours of collection and stored at −80°C. HIPAA compliant, de-identified samples were shipped frozen on dry ice to SomaLogic from the study centers and stored at -80°C. Samples were thawed once for aliquoting prior to proteomic analysis.

### Sample Blinding

In order to prevent potential bias, this study followed a strict standard operating procedure for sample de-identification and blinding, such that all physical samples and data records were identified exclusively by a unique, unidentifiable barcode number and the key was stored in a secure database accessible only to designated responsible administrators. All sample aliquots run in this study were stored in identical tubes identified only by assigned barcode. The sample blinding code was broken only according to the pre-specified analysis plan for the purposes of classifier training with the training set and classifier verification with the verification set. For the verification sample set, a unique blinding key was generated and provided exclusively to a third party reader (K.C.), unaffiliated with the study centers or SomaLogic, to score and report the final verification results.

### Proteomic Analysis

Serum samples were analyzed on our proteomic discovery platform as described in Gold et al [5]. Briefly, this technology uses novel DNA aptamers that contain chemically modified nucleotides as highly specific protein binding reagents in a unique multiplexed assay that transforms the quantity of each targeted protein into a corresponding quantity of aptamer, which is quantified with a custom hybridization array. Protein quantities are recorded as relative fluorescent units (RFU), which can be converted to concentrations with standard curves. The platform is highly automated [26] and scalable to accommodate a broad range of sample throughput. In this study, 813 protein targets were measured in 15 µL of serum for each subject, and all 1,326 sera were analyzed in a continuous process over a period of eight days. Overall, the results are analogous to a little more than 1,000,000 high quality ELISA measurements. Samples were processed in multiple 96-well microtiter plates, and all 1,326 samples were distributed randomly and their identities were completely blinded throughout the proteomic analysis process.

### Biomarker Selection

Biomarkers were selected with a strategy designed to identify analytes with the highest performance in classifying NSCLC cases from controls across all study sites and that were least affected by preanalytical variables. In the first step of this analysis, we eliminated analytes that exhibited unexpected variation compared to internal controls, due to, for example, sample instability. In this process, we chose a set of analytes that performed well in a total of six naïve Bayes (NB) classifier training analyses. First we divided the training set into two distinct populations to control for possible biological variability between them: (1) all cases and controls with benign nodules identified by CT; and (2) all cases and all other smoker controls (nodule status unknown). For each population, we compared cases to controls in three NB training analyses designed to control for potential preanalytical variability between study sites. The three NB analyses started with a unique set of potential biomarkers based on the following criteria: (1) cases versus controls KS≥0.3 for all comparisons within each of the four study sites; (2) cases versus controls KS≥0.3 for comparing all sites combined; (3) both criteria one and two were met. For each analysis, we used a greedy forward search algorithm to select subsets of potential biomarkers, build NB classifiers (see below), and scored their performance for classifying lung cancer and controls using the training set. In this process, this meta-heuristic approach efficiently searches classifier space to identify potential biomarkers that perform best in classification. We used a simple measure of diagnostic performance of classifiers, the numerical sum of sensitivity + specificity, and measured the frequency with which potential biomarkers were selected by the greedy algorithm for inclusion in classifier panels with sensitivity + specificity ≥1.7. This step produced a set of potential biomarkers for each of the six parallel analyses. We selected the final set of biomarkers as the union of these six sets.

### Statistical Methods

The KS statistic is a non-parametric measure of the difference between two distributions. The two-sample KS Statistic is: 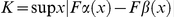, where 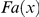
and 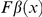
are empirical cumulative distributions for two populations of values.

The naïve Bayes classifier assumes independence between the samples, and models the distributions of the training classes to make predictions [27]. We used normal distributions to model our data. However, the features in our data often contain distributions with heavy tails so maximum likelihood estimation of the distribution parameters performs poorly. Therefore, we modeled our distributions as log-normal distributions and used the Gauss-Newton algorithm to fit the data.

We constructed Bayesian classifiers using sets of potential biomarkers identified as described above. We used a parametric model to capture the underlying protein distribution for a given state. The simplest parametric model for the probability density function (pdf) for a single protein is a normal distribution, completely described by a mean u and variance σ^2^ (Eq. 1).
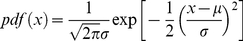
(1)


Many protein distributions were observed as normal with respect to the logarithm of the concentration. The numeric cdfs can be fit to a normal distribution in log concentrations x (Eq. 2).
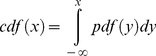
(2)


The models fit the data well. More complex models of the probability distribution functions may be used when warranted but the simple model provided a good description of our data.

To combine multiple markers, we used a multivariate normal distribution to model the probability density function (pdf) for each class. For n markers, the multivariate pdf is given by the following equation (Eq. 3).
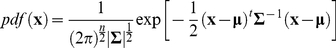
(3)


where **x** is an n-component vector of protein levels, µ is an n-component vector of mean protein levels, Σ is the n x n covariance matrix and |Σ| and Σ^−1^ are its determinant and inverse. In its simplest form, we can assume a diagonal representation for Σ. Such an approximation leads to a naïve Bayes model, which assumes independence between the markers. In this work, we exclusively use the naïve Bayes model for constructing classifiers. The parameter values for µ and Σ used in the naïve Bayes classification were obtained from nonlinear regression analysis as described above.

The addition of subsequent markers with good KS distances will, in general, improve the classification performance if the subsequently added markers are independent of the first marker. We searched for optimal marker panels with a “greedy” algorithm, which is any algorithm that follows the problem solving meta-heuristic of making the locally optimal choice at each stage with the hope of finding the global optimum. We used the sensitivity (fraction of true positives) plus specificity (fraction of true negatives) as a classifier score. The algorithm approach used here is described as follows. All single analyte classifiers were generated from a table of potential biomarkers and added to a list. Next, all possible additions of a second analyte to each of the stored single analyte classifiers were performed, saving a predetermined number (10,000 in this case) of the best scoring pairs on a new list. All possible three marker classifiers are explored using this new list of the best two-marker classifiers, again saving the best thousand of these. This process continues until the score either plateaus or begins to deteriorate as additional markers are added.

## Results

We analyzed 1,326 serum samples from four independent biorepositories: New York University (NYU) [24]; Roswell Park Cancer Institute (RPCI) [25]; The University of Pittsburgh (PITT) [5]; and a commercial biorepository (BioServe (BS)) (Table 1). The study included patients diagnosed with pathologic or clinical stage I-III NSCLC and a high-risk control population with a history of long-term tobacco use, including active and ex-smokers with ≥10 pack-years of cigarette smoking (Table 2 and 3). The control populations were selected randomly within each study to represent the patient population at risk for lung cancer that would be candidates for CT screening, with a ratio of case to control of 1 to 3.5.

Samples were randomly distributed into segregated sets for classifier training and verification (Figure 1) with no significant differences in demographics between these sets (Table 2). More than 45% of NSCLC cases were pathologically confirmed stage IA or IB or clinical stage I with adenocarcinoma representing the major histological diagnosis (Table 3). All lung cancer patients had a biopsy-proven cancer diagnosis.

We measured the quantity of 813 proteins in each of the 1,326 samples with our proteomic discovery platform [18]. We followed a pre-specified two-phase analysis plan to identify biomarkers and develop a classifier to distinguish lung cancer subjects from controls within the training set (training phase) and to verify the classifier performance with the blinded independent verification set (verification phase). The training phase entailed two steps – biomarker selection and algorithm training with cross-validation.

To select biomarkers we performed a systematic analysis that narrowed the potential biomarker field for algorithm training to increase the probability of true discovery, yet still cast a relatively broad net. We used a naïve Bayes (NB) method to systematically assess potential biomarker performance with pre-specified criteria. We applied the NB method to subsets of the training data to broaden our cast for potential biomarkers (see Methods). The results identified a set of 44 potential biomarkers (Table 4) that distinguish lung cancer from controls across a range of comparisons in the training set while minimizing potential preanalytical variability – artifacts introduced by variations in sample collection and storage (see below) [28], [29].

**Table 4:** Potential NSCLC biomarkers§.

****#****	Protein Name	UniProt ID	KS	q-value	NB Freq
1	BCA-1	O43927	0.34	2.51E-17	1
2	BMP-1	P13497	0.35	3.49E-18	10
3	C1s	P09871	0.29	3.92E-13	1
4	C9	P02748	0.41	1.33E-24	6
5	Cadherin-1	P12830	0.32	1.47E-15	206
6	Calpain I	P07384 P04632	0.4	8.46E-24	72
7	Catalase	P04040	0.32	1.21E-15	2
8	CD30 Ligand	P32971	0.28	1.22E-12	51
9	CDK5/p35	Q00535 Q15078	0.27	1.34E-11	31
10	CK-MB	P12277 P06732	0.33	2.51E-16	19
11	Contactin-5	O94779	0.29	1.67E-13	3
12	Endostatin	P39060	0.28	8.48E-13	33
13	ERBB1	P00533	0.46	6.32E-31	136
14	FGF-17	O60258	0.31	6.12E-15	6
15	FYN	P06241	0.13	5.19E-04	14
16	HSP 90α	P07900	0.51	7.86E-37	85
17	HSP 90β	P08238	0.39	1.50E-22	7
18	IGFBP-2	P18065	0.36	1.87E-19	54
19	IL-15 Rα	Q13261	0.29	2.62E-13	4
20	IL-17B	Q9UHF5	0.28	1.07E-12	1
21	Importin β1	Q14974	0.4	1.31E-23	30
22	Kallikrein 7	P49862	0.31	1.79E-14	43
23	LDH-H 1	P07195	0.3	8.64E-14	3
24	Legumain	Q99538	0.28	2.52E-12	1
25	LRIG3	Q6UXM1	0.34	1.13E-17	25
26	Macrophage mannose receptor	P22897	0.37	6.21E-21	21
27	MAPK13	O15264	0.34	4.66E-18	1
28	MEK1	Q02750	0.29	2.62E-13	5
29	MetAP2	P50579	0.44	3.40E-28	7
30	Midkine	P21741	0.11	1.67E-03	7
31	MIP-4	P55774	0.29	2.69E-13	43
32	MIP-5	Q16663	0.31	1.53E-14	27
33	MMP-7	P09237	0.38	1.67E-21	36
34	NACα	Q13765	0.33	7.57E-17	5
35	NAGK	Q9UJ70	0.37	1.25E-20	5
36	Pleiotrophin	P21246	0.29	5.02E-13	107
37	PRKCI	P41743	0.41	3.81E-25	97
38	Renin	P00797	0.25	1.69E-10	2
39	RGM-C	Q6ZVN8	0.27	5.43E-12	84
40	SCF sR	P10721	0.35	6.97E-19	107
41	sL-Selectin	P14151	0.29	7.88E-13	57
42	Ubiquitin+1	P62988	0.33	4.09E-17	1
43	VEGF	P15692	0.29	5.47E-13	1
44	YES	P07947	0.28	1.73E-12	47

§ Measure of the relative importance of potential biomarkers selected with KS distance (KS), KS FDR-corrected q-value (q-value), frequency for naïve Bayes (NB Freq),

**Figure 2:**
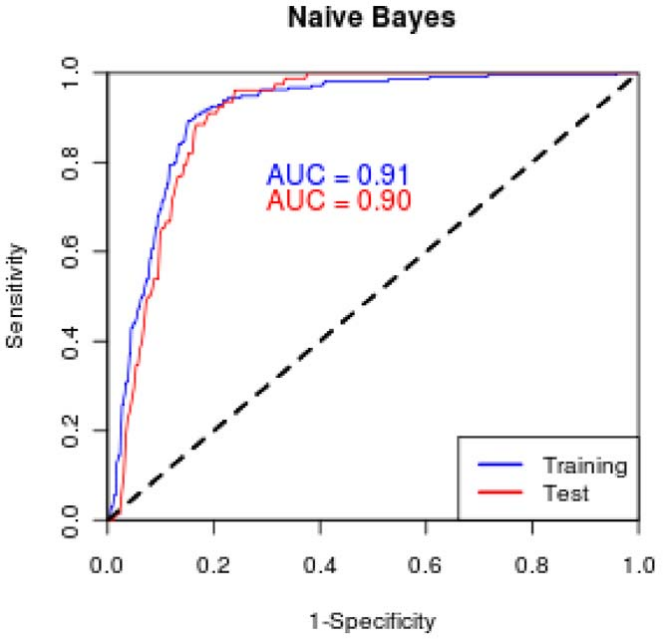
ROC curve for 12-biomarker naïve Bayes classifier.

**Figure 3:**
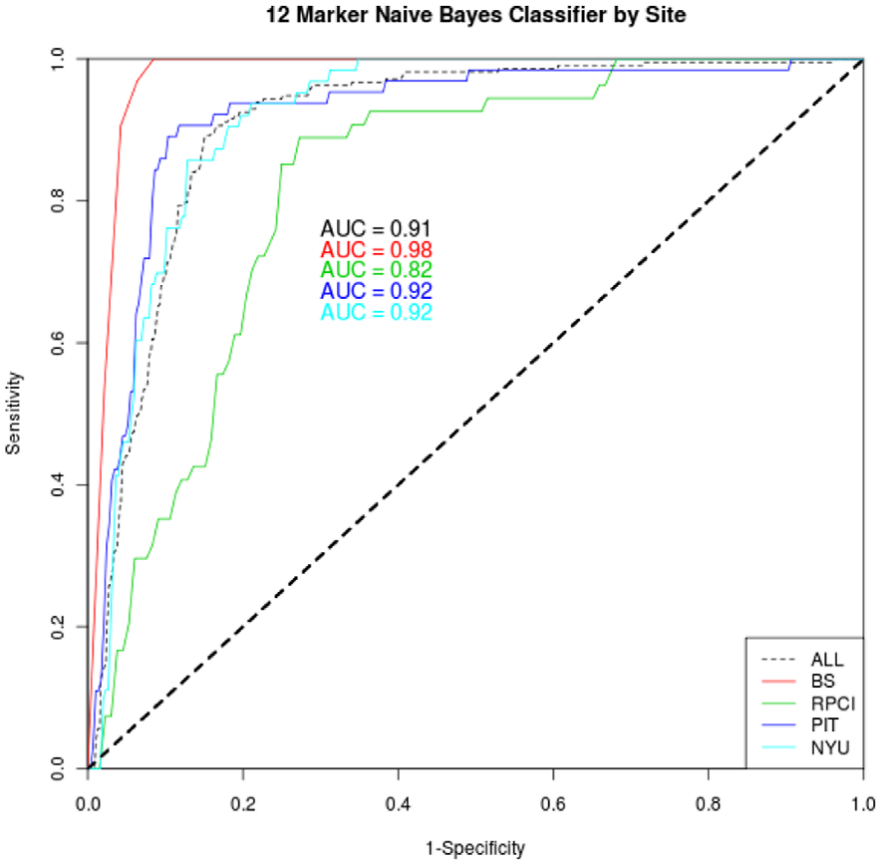
ROC curve performance of the 12-biomarker naïve Bayes NSCLC classifier by study site.

To develop a potential diagnostic to distinguish NSCLC from controls, we trained NB classifiers starting with the 44 potential biomarkers we identified using a “greedy” forward search algorithm and ten-fold stratified cross validation, starting with three biomarkers and adding one more at each step. We assessed classifier performance with pre-specified performance criteria (Table 5). We constructed 45 seven to twelve-biomarker classifiers from this set of 44 potential biomarkers that met our performance criteria, which suggests that there is significant redundancy in the information contained within the set of potential biomarkers. Cross-validated classifier performance reached a performance plateau with twelve biomarkers. Following our analysis plan, we selected from the 45 resulting classifiers one with the highest overall performance of pre-specified criteria (Table 5), including discrimination of NSCLC from controls, detection of Stage I disease, and detection of cancer in chronic obstructive pulmonary disease (COPD). In the training set, the classifier achieved 91% sensitivity, 84% specificity, and an area under the curve (AUC) of 0.91 (Figure 2). The results (Table 6) show that sensitivity is maintained for Stage I NSCLC (90% for training set). The classifier performed well on samples from all four study sites (Figure 3).

**Table 5:** Criteria for algorithm performance on training and cross-validation.

Criteria	Minimum Performance	****#**** Classifiers
Biomarker frequency in greedy algorithm classifiers	10	250
Sensitivity (Stage I-III) + Specificity	1.7	94
Stage I Sensitivity	0.85	80
Cross-validation Sensitivity (Stage I-III)+ Specificity	1.7	50
Cross-validation Stage I Sensitivity	0.85	50
Severe COPD Specificity	0.65	45

**Table 6:** Performance of Bayesian Classifier to distinguish NSCLC cases from controls.

		Sensitivity (%), (95% CI)	Specificity (%), (95% CI)
NSCLC Cases	Training Stage I-III	91 (87-95)	
	Training Stage I	90 (84-96)	
	10-fold Cross Validation	91 (87-95)	
	Verification Stage I-III	89 (81-96)	
	Verification Stage I	87 (78-96)	
Controls	Training All Controls		84 (81-86)
	Training Benign Nodules		82 (78-85)
	10-fold Cross Validation		83 (80-86)
	Verification All Controls		83 (79-88)
	Verification Benign Nodules		85 (79-91)

The twelve biomarkers are shown in Table 7. The estimated serum concentrations for these markers span 4 logs (10 pM-100 nM). About half the control group had benign pulmonary nodules detected by CT (Table 2), and the performance of the classifier in that subgroup was similar to that of the whole (Table 6). We also tested the effect of other attributes that could affect classifier performance such as age, smoking history, and COPD, but found little effect (Tables 8 and 9). Age has a moderate effect on the shape of the ROC curve because the probability of cancer increases with age, but this effect can be controlled by adjusting the prior probability of cancer in the Bayes classifier model. The classification performance of the fixed algorithm was tested on the blinded independent verification set and verified by a third party reader to achieve 89% sensitivity and 83% specificity, nearly matching the training set performance.

**Table 7:** Twelve biomarker classifier proteins§.

Biomarker	UniProt ID	Direction*	Description
Cadherin-1	P12830	down	cell adhesion, transcription regulation
CD30 Ligand	P32971	up	cytokine
Endostatin	P39060	up	inhibition of angiogenesis
HSP 90α	P07900	up	chaperone
LRIG3	Q6UXM1	down	protein binding, tumor suppressor
MIP-4	P55774	up	monokine
Pleiotrophin	P21246	up	growth factor
PRKCI	P41743	up	serine/threonine protein kinase, oncogene
RGM-C	Q6ZVN8	down	iron metabolism
SCF sR	P10721	down	decoy receptor
sL-Selectin	P14151	down	cell adhesion
YES	P07947	up	tyrosine kinase, oncogene

§ Up or down regulation in NSCLC cases relative to controls.

**Table 8:** Performance of classifier in demographic subsets.

		Cases	Controls	Sensitivity (%) (95%CI)	Specificity (%) (95%CI)	Accuracy (%) (95%CI)	AUC
Age	≤61	57	467	84 (75-94)	89 (86-92)	88 (85-91)	0.91
	>61	156	304	93 (89-97)	76 (71-80)	82 (78-85)	0.89
Smoking Status	Current	54	421	93 (86-100)	86 (83-90)	87 (84-90)	0.91
	Ex	85	310	91 (84-97)	85 (80-89)	86 (82-89)	0.93
Pack Years	≤40	84	381	91 (84-97)	86 (83-90)	87 (84-90)	0.93
	>40	76	347	97 (94-100)	84 (81-88)	87 (84-90)	0.94

**Table 9:** Classifier specificity by level of airflow obstruction.

Airflow Obstruction§	FEV1 % Predicted	Number of Patients	Specificity (%), (95% CI)
GOLD 0/I	>80%	411	89 (86-92)
GOLD II	50–80%	167	84 (78-89)
GOLD III/IV	<50%	32	72 (56-87)

§ Spirometric classification of airflow obstruction based on GOLD staging [60].

To determine whether our classification results were affected either by age, smoking status, or smoking history, which are the demographics with significant differences between the case and control populations (Table 2), we compared the classifier performance on subsets of the training set population divided into groups based on the median value of these attributes. The results show similar classifier performance for all subsets (Table 8). To further assess whether our classification results were affected either by age, smoking status, or smoking history, we tested for potential correlation of the twelve biomarkers with these variables. The results showed no correlations except for endostatin, which showed a moderate correlation, increasing with age. This effect can be compensated for by adjusting the prior probability of cancer in the Bayes classifier model. We also assessed the specificity of the classifier for the discrimination of controls known to have airflow obstruction (measured by GOLD score). The results are shown in Table 9. Spirometry data was incomplete for NSCLC cases, so we could not calculate sensitivity.

**Figure 4:**
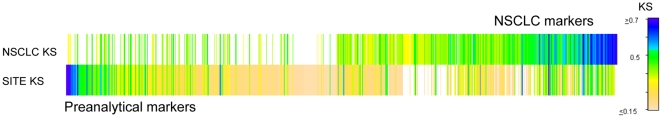
Heat map shows the magnitude of difference for each protein measured (columns) between subject populations for the comparison of NSCLC to controls (top row) and comparisons of cases or controls between study sites (bottom row). Top row: KS distances for NSCLC versus control distributions. Bottom row: mean KS distances for all 12 pair-wise comparisons, between the four sites, of case and control samples analyzed separately. Proteins were ordered by subtracting the NSCLC KS distance from the mean site KS distance. This revealed groups of NSCLC biomarkers (top right) contrasting with preanalytical markers (bottom left).

Preanalytical variability underlies common failures to translate candidate biomarkers into clinically useful tests [20], [29]. We assessed preanalytical variability in this study by measuring differences in protein levels within the same disease class (NSCLC or control) between different sites and comparing them to differences observed between NSCLC and control populations. The results (Figure 4) show significant preanalytical variability between sites. However, proteins most affected are distinct from potential NSCLC biomarkers. Many proteins that exhibit preanalytical variability (Table 10) are known to be susceptible to variations in sample collection and handling [28], [29]. This result confirms that pre-analytical variability exists in our study and provides evidence that, as designed, our study largely overcomes this variability to maximize the chances of discovering true, robust biomarkers of NSCLC.

**Table 10:** Proteins with Preanalytical Variability§.

Protein	UniProt ID	Avg. KS
C3	P01024	0.7
C3a	P01024	0.71
C3adesArg	P01024	0.64
iC3b	P01024	0.66
C3b	P01024	0.59
BMP-14	P43026	0.45
Coagulation Factor IXab	P00740	0.42

§ Proteins that exhibited an average KS distance ≥0.4 for within-site, class-dependent comparisons of preanalytical variation as shown in Figure 4 and that have been identified independently as subject to significant variations due to sample handling in serum.

## Discussion

The primary findings of this study are 44 potential lung cancer biomarkers that discriminate stages I-III NSCLC cases from at-risk heavy smoker controls that can be combined into classifier panels that meet and exceed pre-specified performance criteria. The results of this study are novel in the following: (1) most of the proteins identified in this study have not been identified previously as serum lung cancer biomarkers; (2) we have identified novel protein biomarker panels that distinguish lung cancer cases from appropriate controls with high sensitivity and specificity in an independent, blinded verification set; and (3) this study achieves a new level of evidentiary standard in clinical proteomic biomarker studies as a result of a large sample size, a study design to control preanalytical variability, and the unique capability of this proteomic technology to interrogate the circulating proteome quantitatively with a breadth, sensitivity, and dynamic range unmatched by other broad serum profiling platforms [18], including mass spectrometry [18], antibody arrays [18], and autoantibody arrays [18], [30]–[32]. This study is the first large-scale application of this technology and the largest clinical proteomic biomarker study to date. As such, this study aims to overcome critical confounders and limitations of clinical proteomic biomarker studies that contribute largely to the lack of translation to the clinic due to false discovery [20]. These confounders and limitations include clinical sample integrity, preanalytical variability, and inadequate study design and power.

The best overall performing classifier used 12 of the 44 biomarkers and achieved 91% sensitivity and 84% specificity in cross-validated training and similar performance of 89% sensitivity and 83% specificity in blinded validation. These results provide evidence that these biomarkers are valid and that the classifier was not over-fit to the training data. This performance and the biological plausibility (following) of the 12 biomarkers are encouraging for the next phase of development – validation in an independent clinical study.

The 12 biomarkers identified in this study (Table 4) encompass functions of cell movement, inflammation, and immune monitoring that may contribute to cancer development. Most of the 12 proteins have been associated generally with cancer biology, some have been identified as candidate lung cancer biomarkers, none have been validated as lung cancer biomarkers, and none are used clinically [33], [34]. Four of the 12 proteins have been identified in serum and lung cancer tissue or cell culture as candidate lung cancer biomarkers – cadherin-1 [35], endostatin [36], HSP90 [37], and pleiotrophin [38]. Eight of the 12 proteins, CD30 ligand, LRIG3, MIP-4, PRKCI, RGM-C, SCF-sR, sL-Selectin, and YES, have not been identified previously in serum as lung cancer biomarkers and represent novel findings.

Six of the 12 proteins, CD30 ligand, endostatin, HSP90, MIP-4, pleiotrophin, PRKCI, and YES were observed up-regulated in lung cancer in this study, consistent with their proposed biological roles in proliferation, invasion, or host inflammatory and immune response to the tumor. CD30 ligand is a member of the TNF ligand superfamily, which stimulates T-cell growth. Up-regulation of this protein correlates with proliferation in hematological malignancies [36]. Endostatin, best known as an inhibitor of angiogenesis, has elevated serum levels in several cancers [39]. Overexpression of endostatin and its parent extracellular matrix protein, collagen XVIII have been associated with poor prognosis in NSCLC [36].

The chaperone HSP90α is important for the stability of and function of a wide range of oncoproteins, including BCR-ABL, ERBB2, EGFR, BRAF, and AKT, among others, and inhibitors of this protein are now in oncology clinical trials, including NSCLC [40]. HSP90 may also play a role in tumor cell resistance to complement mediated cytotoxicity [41]. MIP-4 is over-expressed in ovarian and gastric cancers, and may have a role in immunosuppression of the host tumor response [42]. Pleiotrophin is a growth factor with both mitogenic and angiogenic properties and levels in the serum of NSCLC patients have been reported to correlate with disease stage and prognosis [38]. PRKCI is an oncogene that is often amplified in NSCLC and over-expressed in lung tumors correlates with poor prognosis [43]. YES, another protein kinase and member of the src-family of tyrosine kinases, has a role in malignant transformation and increased protein levels have been reported in early stages of hepatocarcinoma [44].

We observed decreased levels of proteins in the serum of lung cancer patients compared to controls, including cadherin-1, LRIG3, sL-selectin, SCRsR, ERBB1 and RGM-C. Lower circulating levels of many of these proteins are associated with relief of inhibition of growth and invasion. For example, cadherin-1 is critical for cell adhesion and indirectly affects transcriptional regulation circuits through β-catenin [45]. Consistent with our results, reduced expression has been reported in lung cancer, and loss of cadherin-1 is a key event leading to loss of adherence, tumorgenicity, and metastasis [46]. The LRIG family consists of membrane proteins with soluble leucine rich repeat domains and immunoglobulin-like domains. Down-regulation of expression of this protein in glioblastoma cell lines resulted in increased proliferation and invasion, decreased apoptosis, and increased EGFR expression, leading to the hypothesis that LRIG is a tumor suppressor [47]. L-selectin plays a role in activation of naïve lymphocytes that participate in immune surveillance and antitumor immunity. It also mediates the adherence of lymphocytes to endothelial cells. Lower expression of L-selectin may be a component of the immune suppression observed in many cancer patients [48].

Some of the proteins described in this study are the soluble domains of membrane receptors, and the function of the circulating form of these proteins may oppose their membrane-bound counterparts. Turner et al. [49] proposed that soluble SCF-receptors regulate kit activation. Our results suggest that a low level of SCF-sR fails to titrate SCF, which makes more SCF available for binding cancer cells. Unlike the membrane bound form, soluble RGM-C inhibits hepcidin expression [50], [51]. We find that RGM-C is down regulated in NSCLC serum, consistent with increased intracellular iron and proliferative cell growth [52].

The limitations of this study include the following. We did not test cases prior to clinically apparent disease. We did not demonstrate organ-specificity and many of the markers are known to be elevated in other cancers. However, the markers will be used in combination and in the proper diagnostic context, such as with imaging, smoking history, and symptoms. We did not validate our findings in an independent set of clinical samples. Our multi-center study was designed to minimize the effects of potential preanalytical variability, which is mitigated, but not eliminated by this study. All of these limitations will be addressed in the next phase of development, which is enabled by the positive results of this study.

The biomarkers that we discovered have several potential clinical applications. The first application is early detection of lung cancer in long-term smokers when it may be cured by surgery. Our results are a significant improvement on the performance of other recently published lung cancer biomarker studies aimed at early diagnosis [17] using mass spectrometry [24], [53], [54] or gene expression [55]. This performance could allow for testing of individuals with increased lung cancer risk, with subsequent CT screening based on the blood test result.

A second potential application is a test for diagnosing lung cancer in subjects with suspicious lung nodules identified by CT, which could help mitigate the problem of morbidity and cost associated with surgical interventions. CT screening reveals suspicious nodules in ∼40% of long-term smokers [5], [56], [57], but ∼97% are likely benign [5], [57], [58]. Protocols for managing these patients balance the risk of “watchful waiting” with definitive and costly invasive procedures. Watchful waiting monitors nodule growth by periodic follow-up CTs, but may miss the opportunity for early surgical cure. Invasive procedures incur the risk of complications and death that arise from biopsy or futile thoracotomy for benign lesions. This risk might be reduced by a new strategy to assess nodule volume doubling time by CT [13]. However, CT radiation itself increases cancer risk [59].

Based on the discoveries reported here, we have initiated clinical validation studies of populations at risk for lung cancer. Our goal is to develop a clinical blood test to enable an earlier diagnosis. This study is the first to be published in a sequence of successful biomarker discovery studies that we have already completed in different cancers and demonstrates the power of our proteomic technology to discover robust biomarkers in important diseases. This general approach can also be applied to discover biomarkers for many more conditions including infectious, inherited, neurological, and metabolic diseases.
